# A motivational interviewing intervention to PREvent PAssive Smoke Exposure (PREPASE) in children with a high risk of asthma: design of a randomised controlled trial

**DOI:** 10.1186/1471-2458-13-177

**Published:** 2013-02-27

**Authors:** Sasha G Hutchinson, Ilse Mesters, Gerard van Breukelen, Jean WM Muris, Frans JM Feron, S Katharine Hammond, Constant P van Schayck, Edward Dompeling

**Affiliations:** 1 Department of Paediatric Pulmonology, Maastricht University Medical Centre (MUMC+) / CAPHRI School for Public Health and Primary Care, P.O. Box 616, Maastricht, MD 6200, the Netherlands; 2 Department of Epidemiology, MUMC+ / CAPHRI, P.O. Box 616, Maastricht, MD 6200, the Netherlands; 3 Department of Methodology and Statistics, MUMC+ / CAPHRI, P.O. Box 616, Maastricht, MD 6200, the Netherlands; 4 Department of General Practice, MUMC+ / CAPHRI, P.O. Box 616, Maastricht, MD 6200, the Netherlands; 5 Department of Social Medicine, MUMC+ / CAPHRI, P.O. Box 616, Maastricht, MD 6200, the Netherlands; 6 School for Public Health, University of California, 50 University Hall 7360, Berkeley, CA 94720-7360, USA

**Keywords:** Children, Asthma, Passive smoke exposure, Motivational interviewing, Intervention

## Abstract

**Background:**

Especially children at risk for asthma are sensitive to the detrimental health effects of passive smoke (PS) exposure, like respiratory complaints and allergic sensitisation. Therefore, effective prevention of PS exposure in this group of vulnerable children is important. Based on previous studies, we hypothesized that an effective intervention program to prevent PS exposure in children is possible by means of a motivational interviewing tailored program with repeated contacts focussing on awareness, knowledge, beliefs (pros/cons), perceived barriers and needs of parents, in combination with feedback about urine cotinine levels of the children. The aim of the PREPASE study is to test the effectiveness of such an intervention program towards eliminating or reducing of PS exposure in children at risk for asthma. This article describes the protocol of the PREPASE study.

**Methods:**

The study is a one-year follow-up randomized controlled trial. Families with children (0–13 years of age) having an asthma predisposition who experience PS exposure at home are randomized into an intervention group receiving an intervention or a control group receiving care as usual. The intervention is given by trained research assistants. The intervention starts one month after a baseline measurement and takes place once per month for an hour during six home based counselling sessions. The primary outcome measure is the percentage of families curtailing PS exposure in children (parental report verified with the urine cotinine concentrations of the children) after 6 months. The secondary outcome measures include: household nicotine level, the child’s lung function, airway inflammation and oxidative stress, presence of wheezing and questionnaires on respiratory symptoms, and quality of life. A process evaluation is included. Most of the measurements take place every 3 months (baseline and after 3, 6, 9 and 12 months of study).

**Conclusion:**

The PREPASE study incorporates successful elements of previous interventions and may therefore be very promising. If proven effective, the intervention will benefit the health of children at risk for asthma and may also create opportunity to be tested in other population.

**Trial registration number:**

NTR2632

## Background

Despite current efforts to prevent passive smoking, some people are still being exposed to passive smoke (PS) involuntarily, especially children because they cannot protect themselves. The World Health Organization estimates that about 40% of children worldwide are exposed to PS [1]. Currently, about 25% of the Dutch population 15 years and older smoke [2]. Although we have seen a major decrease in PS exposure in children, children with (heavy) smoking parent(s) and children from socially deprived families are more likely to be exposed [3]. The harmful health effects of PS exposure in children are enormous and include higher risks of respiratory complaints, varying from airway infections like bronchitis and pneumonia, allergic sensitization to allergens and asthma, to even more serious events like sudden infant dead syndrome and meningococcal septic shock [4-7]. Furthermore, the effect of PS exposure on the airways in children is modified by a positive family history of asthma [8,9]. This underlines the importance of effective strategies to prevent PS exposure in children, particularly those with high risk of asthma. The available literature clearly indicates that prevention of PS exposure in children is difficult to achieve. Various intervention strategies have been developed and tested, but the results are mixed. A narrative review of the effectiveness of 36 trials found only 11 trials to be successful in reducing PS exposure in children [10]. However, 23 of the 36 studies showed an overall reduction of PS exposure in children regardless of the group allocation. A more recent review and meta-analysis of 18 trials found a positive intervention effect in 13 studies, with 4 showing a statistically significant advantage for the intervention group [11]. Overall, the trial designs were diverse, ranging from a clinical setting to home-based, different trial durations, and counselling modalities extending from personal behavioural to web-based counselling. Yet, certain elements of intervention studies appeared to be effective. Positive effects on stopping PS exposure in children have been described for behavioural counselling methods, such as motivational interviewing (MI) [12]. MI is a client-centred, directive method for enhancing intrinsic motivation to change by exploring and resolving ambivalence [13]. In a scientific setting, MI outperformed traditional advice in the treatment of a broad range of behavioural problems and diseases [14]. But behavioural counselling alone is probably not enough for an effective, long-lasting intervention effect to prevent PS exposure in children [15,16]. The addition of repeated feedback on children’s urinary cotinine levels to behaviour-changing strategies seemed to reduce the urine cotinine concentrations in children and the proportion of parents quitting smoking [17,18]. Also, brief interventions, even when used with additional information brochures, were not effective [19,20]; indicating that more than one counselling session is needed to increase the effectiveness of interventions towards stopping of PS exposure in children. The development of an effective intervention program to prevent PS exposure in children is challenging. However, an intervention program that incorporates all successful elements of earlier studies may have high chance of being effective.

### Hypothesis

The hypothesis of the PREPASE study (PREvention of PAssive Smoke Exposure) is that an effective intervention towards eliminating or reducing PS exposure in children with a high risk of asthma is possible by means of a motivational interviewing tailored program with repeated contacts focussing on awareness, knowledge, beliefs (pro/cons), perceived barriers and needs of parents, in combination with feedback about urine cotinine levels of the children.

### Aim

The aim of the PREPASE study is to test the effectiveness of an intervention program towards eliminating or reducing PS exposure in children with high risk of asthma.

### Our primary research question is

What is the effectiveness of a motivational interviewing tailored program including urinary cotinine feedback towards eliminating or reducing PS exposure in children with a high risk of asthma (measured 6 months after the start of the intervention)?

In this study, children with a high risk of asthma are defined as children with a positive family history of asthma in the first degree (father/mother/sibling). The children do not have an asthma diagnosis themselves. The intervention is successful if the percentage of parents reporting to eliminate or reduce PS exposure in their children (also checked with the children’s urine cotinine concentration) 6 months after the start of the intervention is significantly higher in the intervention group than in the control group.

### Our secondary research questions are

1. Does eliminating or reducing PS exposure in children persist after the intervention program (measured at 9 and 12 months of follow-up)?

2. What is the effect of the intervention on parents’ active smoking? Will the intervention also lead to smoking cessation?

3. What is the influence of PS exposure in children with high risk of asthma on their lung function, inflammatory markers in exhaled breath and respiratory complaints?

The aim of this article is to describe the PREPASE study design, which could benefit others who are planning comparable intervention programs.

## Methods/design

### Study design

The study is a one year randomized controlled intervention study comparing “care as usual” to an intervention program towards eliminating or reducing PS exposure in children with high risk of asthma delivered by trained research assistants (RA). The study is conducted in South Limburg, Netherlands at the Maastricht University Medical Centre (MUMC+).

### Eligibility

Inclusion criteria to participate in the PREPASE study are families where the youngest child is 0–13 years old and he/she does not has an asthma diagnosis but has a high risk of asthma (father/mother/sibling has asthma) and is exposed to PS at home by at least one parent. Exclusion criteria are: children who are actively smoking themselves, parents and/or children with mental retardation, children with lung diseases such as asthma, or cystic fibrosis, and, families already receiving professional support for smoking cessation.

### Recruitment

#### Self-referral strategies

First, a random sample of 6987 children aged 0–13 years are selected from the community registries of the civil affairs department of three cities (Heerlen, Maastricht and Sittard-Geleen) in South Limburg. Second, 3013 children aged 0–13 years are selected form the Registration Network of Family Practices (RNH Dutch acronym) [21]. All families receive an invitation letter to participate in the study, including an informed consent form, two questionnaires (A and B) and a stamped addressed envelope. Parents who want to participate are instructed to complete questionnaire A for their youngest child and return it to the research group with the informed consent form. Parents who do not want to participate are asked whether they are willing to complete questionnaire B for the purpose of a non-response analysis. Parents are informed that participation is voluntary but in case of no response, a single reminder letter is sent after two weeks. Questionnaire A includes 91 items (see Additional file 1) and is composed of three parts. The first part consists of general questions on family characteristics: gender and birth date of the child, relationship of the caregiver(s) to the child, birth date of the caregiver(s), the number and birth date of siblings, education level and working situation of the caregiver(s), and ethnicity. The second part of the questionnaire inquire about the child’s general and respiratory health using a Dutch version of the ISAAC questionnaire [22,23], and questions concerning physician diagnosed respiratory infections in the past 12 months, vitamin use, gestational age at birth, birth weight, complication(s) during pregnancy, breast feeding, smoking during pregnancy, diagnosis of syndrome(s) or congenital disease(s), day-care attendance, and the presence of physician diagnosed asthma, eczema or hay fever in the biological parents and or siblings of the child. The third part of the questionnaire consists of questions about parental smoking behaviour and passive smoke exposure towards the child. Smoking behaviour of the parents is measured with a standard questionnaire [24]. Questions about PS exposure in children are derived from earlier studies [25,26] and expert opinions from researches in the field, and included sources of PS exposure at home and elsewhere. Questionnaire B consists of 11 items, including gender and birth date of the child, relationship of the caregiver(s) to the child, wheezing and respiratory complaints in the past 12 months, physician diagnosed asthma, PS exposure during pregnancy and currently, and reasons for not participating in the study.

The third source of recruitment is via cohorts from the MIKADO study [27] and the ADEM study [28] from the department of child pulmonology MUMC+. Families that do not meet the eligibility criteria for the MIKADO study or who complete the ADEM study, and in both cases, give agreement to be contacted for other studies receive a letter from the MIKADO or ADEM team asking them if they would like to be contacted to receive information from the PREPASE study.

The fourth self-referral recruitment strategy is done through primary schools in South Limburg. Parents of 32.000 children aged 6–12 years receive an information letter to participate in an electronic survey (see Additional file 1) study regarding respiratory complaints in children in South Limburg. All letters include the web address of the survey and a unique password to log into the website. The electronic questionnaire is similar to questionnaire A, but with minor adjustments. From the ISAAC only questions regarding wheezing and asthma are used. Moreover, questions regarding parental smoking behaviour are limited to PS exposure towards the child. Schools are encouraged to motivate participation. After two weeks, all parents receive a reminder letter through the primary schools of their children. The fifth source of recruitment is through advertisements in local newspapers.

#### Physician based strategies

Since our target group is likely to consist of a more disadvantaged or vulnerable population, physicians working in the paediatric field (general practitioners, paediatricians and doctors of child and youth health care) are asked to select eligible families to participate from their patient registries or actively during consultations [29]. These families receive information from the study through their physicians and are asked by their physicians if they grant permission to be contacted by a PREPASE study team member for more information. Furthermore, registries of all children in South Limburg at the Regional Public Health Service (GGD Dutch acronym) are checked for eligibility (child 0–13 years of age with high risk of asthma and PS exposure at home). Eligible families receive an invitation letter from their physician to participate in the study.

Except for the recruitment through the community registries and RNH, we cannot control if a family is approached more than once. For the analysis, recruitment source is taken into account. Regardless of the recruitment method, all parents are informed that the MUMC+ is testing the effectiveness of a program to prevent PS exposure in children with high risk of asthma, that the program occurs in the home setting and includes non-invasive measurements of the lung function of the children and that all families will receive a financial reward after complete participation. Parents can give permission to be contacted by a member of the research group if they want more information or are interested to participate in the study.

### Randomisation

**Figure 1:**
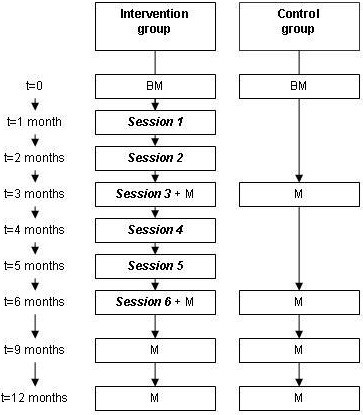
**Flowchart of the PREPASE study.** BM = baseline measurement. M = Measurement. The first session starts 1 month after the BM and take place once per month during 6 months. The measurements take place every 3 months after the BM.

Eligible families are contacted by phone to ensure eligibility and willingness to participate in the study. Eligible families, after giving written informed consent, are randomized into one of the two groups, the intervention group or control group, with an allocation ratio of 1 (see Figure 1). A database logistic system is made for the study by MEMIC (Centre for data and information management, MUMC+), and includes a randomization system. Families are pre-stratified according to the age of the child, <6 years and ≥6 years, to prevent chance imbalances between the intervention and the control group with respect to age and age-related variables, such as lung function and possible parental attitude towards passive smoking (parents with younger children may evaluate PS exposure differently from parents with older children). Separate randomization lists are generated for each stratum to ensure treatment group assignments are balanced within each stratum.

Blinding of participants or members of the research team for this study is not possible. However, prior to randomization the participants receive limited information on the intervention program. Participants are informed that the study aims at testing a program to help parents prevent PS exposure in their children and to study the relationship between PS exposure and respiratory complaints in children.

After randomization, parents receive more information about their group allocation. All visits are planned for the entire study period, and a confirmation letter is sent to the participants including information and materials with instructions for the urine sampling and lung function measurements in children.

### Intervention program towards eliminating or reducing of PS exposure in children

In MI, the parents’ choice, personal responsibility for change, and enhancement of self-efficacy are emphasized [13]. The communication method (using open-ended questions, providing affirmation, listening reflectively and providing summaries) elicits the parents own inherent arguments for change (change talk), and increases their awareness of the discrepancy between their current behaviour and what would happen if they decide to eliminate or reduce PS exposure in their children. Counselling is focused on building motivation within parents, empathy and giving confidence to parents. Possible ambivalence with the decision to eliminate or reduce PS exposure in their children (and to stop active smoking) is discussed. Goal setting is used to help parents identify steps that they want to take to prevent PS exposure in their homes. Possible barriers experienced by the parent(s) are also discussed. As an awareness raiser, parents receive feedback from the children’s urine cotinine concentrations. The intervention program is based on the principles of the reasoned action model for the prediction and change of behaviour [30]. This model suggests that behaviour is best predicted by a person’s intention to perform certain behaviour. The intention to perform behaviour is predicted by a combination of considerations which take on different weights. These considerations are formed by a person’s: 1) beliefs towards the behaviour which determines their attitude towards the behaviour, 2) normative beliefs concerning the behaviour which produce a perceived norm concerning the behaviour, in other words, the perceived social pressure to (not) engage in a certain behaviour, and 3) control beliefs which are the person’s perceived personal and environmental factors that can facilitate or inhibit performance of the behaviour. Moreover, background factors such as past behaviour, education, and abilities also influence a person’s beliefs and resulting behaviour. These behavioural determinants are measured and considered during the sessions of the PREPASE intervention program. The intervention is given by two trained RAs and consists of 6 counselling session at home during 6 months, each lasting about 60 minutes (see Figure 1). Each family is coupled with one of the RAs. The RAs are prepared by receiving a one day training “Growing up Smoke Free (Training rookvrij opgroeien)” given by an expert from STIVORO (Dutch expert centre on tobacco control). The training includes general information regarding smoke addiction, the health effects and measures to prevent PS exposure in children, and basic training on motivational interviewing. Further coaching is given on a regular basis.

In the beginning, parents are told that our primary focus is on preventing PS exposure in children, and that quitting active smoking is not required. The intervention is tailored to whether parents are interested in reducing and/or eliminating PS exposure in their children, or if they are interested in quitting active smoking as well. If both parents of a child smoke, they are counselled together to enable a long-lasting intervention effect. If only one parent smokes, the other parent is still requested to be present during the session. The non-smoker parent may motivate the smoking parent or and reinforce his or her goals to prevent or eliminate PS exposure in their child. If parents permit, the counselling sessions are recorded on audio. The purpose of the recordings is to randomly examine the treatment fidelity. Although the intervention is tailored, a counselling protocol is developed to serve as a guideline for the counselling sessions. The following subjects are discussed:

### Session 1 - awareness of passive smoking in children

1. After an introduction by the RA details of parental smoking behaviour are assessed: number of cigarette smoked per day, possible quitting attempts in the past and the successes in these attempts, smoking behaviour inside the house and in the presence of their children. Afterwards, the parents are informed about PS exposure in children: the hazardous chemicals found in tobacco smoke, and the forms of PS exposure (second-hand smoke and third-hand smoke [31]).

2. Knowledge about the health effects of PS exposure in children is assessed and if parents want more information, this is provided.

3. Motivation and self-efficacy to prevent PS exposure in children are assessed with the importance and confidence ruler developed by Rollnick [32].

4. Parental readiness to prevent PS exposure in children is assessed. In case parents are ready to start preventing PS exposure in their children, the following subjects are discussed: measures to prevent PS exposure in children, parents’ personal goals, and, possible barriers and solutions. They are encouraged to make a ‘quit plan’ (see Table 1), that includes a quit date and their specific measures and goals. In case parents are not ready to change their behaviour, ambivalence is assessed and parents are encouraged to think about the cons and pros of preventing PS exposure in children for the following session.

5. The RA gives a summary of the session.

**Table 1:** An overview of a ‘quit plan’

**Quit plan**	
***My goals are:***	•………………………………………………………‥
	• ………………………………………………………‥
***My most important reason(s) for preventing passive smoke exposure in my child is (are):***	1) ………………………………………………………
	2) ………………………………………………………
***To accomplish my goal(s), I will take the following measures:***	
Specific action:	Date:
1) ……………………	……………………………………………………………
2) ……………………	……………………………………………………………
***These are the following difficulties I may encounter and how I plan to solve them:***	
Difficulties:	Solution:
1) ……………………	……………………………………………………………
2) ……………………	……………………………………………………………
***Other person(s) that could help me achieve my goals:***	
Person(s):	How?:
1) ……………………	1) …………………………………………………………
2) ……………………	2) …………………………………………………………
***I will use the following results to ensure me that I am achieving my goals:***	• ………………………………………………………‥
	• ………………………………………………………‥

A copy of the ‘quit plan’ is included in the information booklet.

### Session 2 - preparation, planning quit-date and plan

1. The RA gives a short summary of session 1.

2. For parents who did not make a plan to quit PS exposure in their child: motivation, self-efficacy and readiness to prevent PS exposure in children are reassessed as described in session 1.3 and 1.4. Possible ambivalence, barriers and solutions are discussed. In case parents are ready, they are motivated to make a ‘quit plan’. Emphasis is put on the positive influence which the parents can have on the health of their children.

3. For parents who made a ‘quit plan’ during session 1 the ‘quit plan’ is evaluated. Self-efficacy is assessed. Difficulties parents may have encountered are also discussed. Parents are encouraged to reward themselves for their efforts. Parents may reformulate their specific goals.

4. Feedback about urine cotinine is given.

5. The RA gives a summary of the session.

### Session 3 - evaluation of the ‘quit plan’

1. The RA gives a short summary of what has been discussed previously (including possible ambivalence, barriers and solutions) and (if applicable) the specific goals parents have made to prevent PS exposure in their children.

2. The ‘quit plan’ is evaluated as described under session 2.3. Furthermore, parents have the opportunity to reformulate their specific goals or add new goals to their ‘quit plan’.

3. For parents who did not make a plan to eliminate or reduce PS exposure in their child: motivation, self-efficacy and readiness to prevent PS exposure in children are reassessed as described in session 1.3 and 1.4.

4. The RA gives a summary of the session.

### Session 4 & 5 - evaluation and focus on difficult situations

1. The RA gives a short summary of what has been discussed previously (session 3.1).

2. The ‘quit plan’ is evaluated as described under session 2.3.

3. Possible difficulties and new barriers are discussed. Parental behaviour towards difficult situations (social influences) such as visiting friends who are accustomed to smoking in the house, are further discussed. Moreover, measures to prevent PS exposure of their children elsewhere, for example when visiting grandparents, are discussed. Parents are motivated to maintain their accomplishments in eliminating or reducing PS exposure in their children. Enhancement of self-efficacy is emphasized. Parents may reformulate their specific goals.

4. Feedback about urine cotinine is given during session 4.

5. The RA gives a summary of the session.

### Session 6 - making long-term goals

1. The RA gives a short summary of what has been discussed previously

2. The ‘quit plan’ is evaluated as described under session 2.3.

3. Possible difficulties and (new) barriers are discussed as described under session 4/5.3.

4. Specific goals for the future (in order to reach a long-lasting intervention effect) are formulated.

5. Feedback about urine cotinine is given.

6. The RA gives a summary of the session and acknowledges the parents for their participation and compliments them for their efforts to prevent PS exposure in their children.

**Figure 2:**
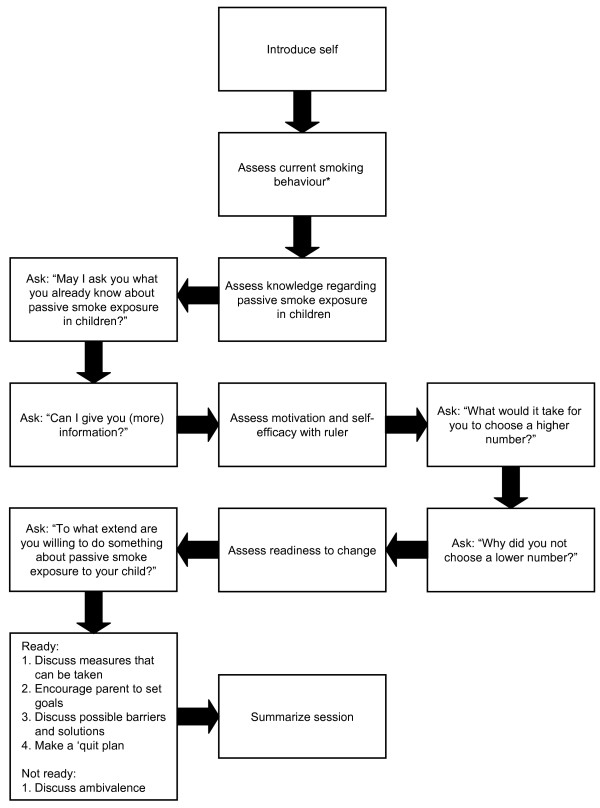
**Schematic presentation of session 1.** *The assessment of current smoking behaviour is done during every session.

The RAs receive an extensive protocol with examples of different scenarios and how to deal with possible resistance during the sessions. Furthermore, to assist the RA during the visits, a summary of each session is given in the form of a schematic presentation; an example of session 1 is given in Figure 2.

Prior to the RCT, the first two sessions of the intervention program were tested and evaluated in 11 parents who reported smoking at home in the presence of their children. Our objective was to test the feasibility of the intervention program and to gain more knowledge on the attitudes and beliefs of parents towards PS exposure in children and the barriers parents may encounter when eliminating or reducing PS exposure in their children. All parents completed the counselling sessions. Before the intervention, 6 parents (54.5%) fully agreed that eliminating PS exposure in their children is of great importance, 2 (33.3%) reported that it would be easy to accomplish. After two counselling sessions, 10 parents (90.9%) were planning to eliminate PS exposure in their children. The pilot study demonstrated good feasibility of the new intervention program. All parents were happy with the intervention style, duration, and found it relevant for parents who are smoking in the presence of their children.

### Control group

During the entire study, the control group receives only measurements according to the same protocol and time plan as for the intervention group. The parents are informed about the results of the measurements after completion of the study. In case parents request more information on passive smoke exposure in children, they are encouraged to contact their general practitioner.

### Outcomes

An overview of the measurements is provided in Table 2. All measurements are home-based and all questionnaires are completed by the parents.

**Table 2:** Overview of the outcome measurements

	***Time (months)***	**0**	**3**	**6**	**9**	**12**
***Outcome measures***						
**Measurements:**						
Urine cotinine		●	●	●	●	●
Household nicotine level		●				●
EBC		●		●		
Wheezometer		●		●		
Long function		●	●	●	●	●
**Questionnaires:**						
Parental smoking behaviour [24], PS exposure to the child Beliefs about PS exposure in children		●	●	●	●	●
Respiratory symptoms and infections (based on questions developed by the PREPASE team and the respiratory symptoms questionnaire [37]		●	●	●	●	●
ISAAC [23]		●				●
Quality of life (FSII) [38]		●	●	●	●	●
Process evaluation						●

The measurements take place in both groups of the RCT, all measurements are home based. The process evaluation questionnaire is completed only in the intervention group. Questionnaires are completed by the parents. PS = passive smoke.

### Primary outcome measures

#### Eliminating or reducing PS exposure in children

PS exposure is measured with a standard questionnaire (at baseline and at 3, 6, 9, and 12 months after the baseline measurement) and is checked by urine cotinine concentrations of the children. In the PREPASE study, the percentage of parents eliminating or reducing PS exposure in children at home at 6 months (directly after the intervention program) is compared between the intervention and the control group. Eliminating or reducing PS exposure in children can be achieved ideally by parents stopping active smoking or less ideally by parents smoking outside the house.

PS exposure in children is measured with the following questions: “Do you smoke in the presence of your child?” (yes/no) If yes, “Where do you smoke in the presence of your child and does this occur always, regularly, sometimes or never?” (Parents are asked to provide an answer for the following locations: living room, own bedroom, child’s bedroom, kitchen, under the hood, hall, dining table/room, attic, rest/bathroom, balcony, garden, car, other) “On average, how many tobacco products do you smoke per day in the house while your child is present at the moment?” (xx cigarettes/cigars/pipe per day) “On average, how many tobacco products do you smoke per day outdoors while your child is present at the moment?” (xx cigarettes/cigars/pipe per day). Furthermore, parental report is verified by the child’s urine cotinine concentration.

#### Urine cotinine of the children

Cotinine is the major metabolite of nicotine and can be detected in several bodily fluids, such as blood serum, saliva and urine [33]. Urine samples are collected (at baseline and after 3, 6, 9, and 12 months after the baseline measurement) and analysed for cotinine using the gas chromatography–mass spectrometry technique, Thermo Scientific DSQIII (Axel Semrau GmbH & Co. KG, Sprockhövel, Germany). Samples from children who have not yet been trained to go to the toilet are collected by placing a highly absorbent cotton wool in the diapers. Samples from toilet trained children are collected with a standard urine collection cup. Early-morning urine samples are collected on the day of the measurements by the RA or primary researcher. The urine samples are analysed at Medical Laboratory Humicon, B.V., Maastricht, the Netherlands. The laboratory receives the urine samples on the same day and stores them at 2-8° Celsius and analyses them within 1–2 weeks. The samples are provided with a numerical ID, the child’s birth year, date and time of collection. The laboratory is blinded to subjects’ identity and group assignment.

### Secondary outcome measures

#### Parental smoking behaviour

Parents active smoking behaviour is measured (at baseline and at 3, 6, 9, and 12 months after the baseline measurement) with a standard questionnaire [24] and questions related to PS exposure in children (in addition to the questions above, parental attitudes, normative and self-efficacy beliefs concerning the prevention of PS exposure in children are also assessed) (see Additional file 1). When applicable, both parents are asked to complete the questionnaire.

#### Household nicotine measurements

Household nicotine levels are measured (at baseline and 12 months after baseline) by placing a passive sampling diffusion monitor in the living room area [34]. The monitors are placed in the homes and removed one week later. The household nicotine concentrations levels are analysed by means of gas chromatography at the University of California at Berkeley School of Public Health.

#### Airway inflammation and oxidative stress

Non-invasive parameters of airway inflammation and oxidative stress are assessed (at baseline and 6 months after baseline) with the handheld exhaled breath condensate (EBC) sampling device Ecoscreen, (CareFusion, Germany). A nose-clip is used and children are instructed to breathe tidally through a mouthpiece with a two-way non-rebreathing valve during 10 minutes. Saliva contamination is prevented by a saliva trap. Children are distracted by watching a video or by playing on a portable gaming device. After 10 minutes the condensate is collected in 1.5 ml Eppendorf tubes (original Eppendorf Protein LoBind Tube). Tubes are filled with 100 μl of condensate, depending on the amount of condensate acquired during collection. Directly after, acidity (pH) of the EBC is measured with pHenomenal pH-1000H (VWR Int., Leuven, Belgium). A minimum of 150-200 μl of EBC is needed for the pH measurement. The Eppendorf tubes are frozen with dry-ice, directly after pH-measurement and are stored at −80 °C. The condensate is analysed for markers relevant for asthma and airway inflammation (IL-4, IL-5, IL-6, IL-8, IL-10, IL-12p70, IL-13 and TNF-α) [35] as described in earlier studies.

#### Wheezing

Possible presence of wheezing is measured (at baseline and 6 months after baseline) with a non-invasive handheld instrument, Wheezometer^TM^ (KaramelSonix Ltd). The Wheezometer is placed behind the manubrium while the child is asked to breathe normally. Breath sounds from the trachea are recorded during 30 seconds and in case wheezing is present, it is presented as a wheeze rate (Tw/Ttot%).

#### Lung function: measuring airway resistance in children ≤5 years of age

In children aged 5 years and younger, measurements of airway resistance are performed (at baseline and at 3, 6, 9, and 12 months after the baseline measurement) by means of the MicroRint (Micro Medical, Rochester Ltd, UK) [36]. Children are asked to sit in an upright position and to breathe normally through a facemask. Their attention is diverted by video clips to guarantee tidal breathing as much as possible. Five airflow interruptions are made on the peak flow of expiration, the median MicroRint value together with the flow and pressure curves are displayed. The median MicroRint value is used for analysis. Afterwards, 300 microgram of salbutamol (Airomir®, Teva Pharma NL, Haarlem, the Netherlands) is inhaled through the AeroChamber® (Trudell Medical International, Ontario, Canada). After 15 minutes, MicroRint measurements are repeated to assess the reversibility to the salbutamol.

#### Lung function: dynamic spirometry in children 6–13 years of age

In children aged 6–13 years, dynamic spirometry is performed (at baseline and at 3, 6, 9, and 12 months after the baseline measurement) by means of the Flowhandy Zan 100USB® (nSpire Health GmbH, Germany). The ZAN-100 pulmonary spirometer is attached to a laptop making it possible to perform home-based measurements. Standard spirometry references are created by entering age, weight, length and sex of the child. Children are instructed and motivated before and during measurement. Younger children, below 7–8 years of age, are stimulated to perform better by using motivational images. A maximum of 8 maximal expiratory flow volume (MEFV) curves are performed, until three technically satisfactory measurements are achieved (ATS/ETS criteria). Thereafter, 300 microgram of salbutamol (Airomir®, Teva Pharma NL, Haarlem, the Netherlands) is inhaled. After 15 minutes, MEFV curves are measured as described earlier to assess the reversibility to salbutamol. The highest forced expiratory volume in one second (FEV_1_), forced vital capacity (FVC), and maximal expiratory flow at 50% FVC (MEF_50_) are used for analysis. The FEV_1_ is expressed as a percentage of the predicted value (gender, age, ethnicity, weight, height, ambient temperature, humidity and barometric pressure taken into account).

#### Questionnaires on respiratory symptoms and quality of life

The presence of respiratory symptoms is assessed with the ISAAC questionnaire [22,23] (at baseline and 12 months after baseline) and a standard questionnaire [37] (at baseline and at 3, 6, 9, and 12 months after the baseline measurement). Furthermore, the quality of life (FSII) [38] in the children is also measured (at baseline and at 3, 6, 9, and 12 months after the baseline measurement). (See Additional file 1).

#### Process evaluation

Parents in the intervention group receive a questionnaire developed by our project group at the end of the study to evaluate their overall experience and the different components of the intervention program: content, counselling style, the RA, and the information booklet (see Additional file 1). Furthermore, random audio samples of the sessions are independently evaluated by two trained raters using the behaviour change counselling index (BECCI) [39]. The BECCI measures the RAs competence in behaviour change counselling. The counselling sessions are rated with the 11 5-point Likert-scale items of the BECCI scale to generate an overall score.

### Statistical methods

#### Sample size calculation

The sample size calculation is based on the primary outcome measure, the percentage of families eliminating or reducing PS exposure in the intervention group versus the control group after 6 months. Assuming a 10% stopping PS exposure rate (by elimination or reduction) in the control group because of a ‘trial effect’, a clinically relevant difference in stopping rate between intervention and control group of 20% (stopping rate of 30% in the intervention group), a two tailed test with an alpha of 0.05 for the primary outcome, and a power of 80%, a total number of N=126 families is necessary, assuming one child is included per included family. With an anticipated 15% drop-out rate in the RCT, at least 148 families (74 per group) are necessary to test the effectiveness of the intervention.

#### Statistical methods

The results of the questionnaires are converted into frequencies and percentages for binary and polytomous variables and averages for continuous variables. For descriptive purposes, baseline summary statistics of demographics and outcomes will be tabulated per group. The effect of the intervention will be assessed by comparing the intervention and control group with respect to the percentage of stopping (by eliminating or reducing) of PS exposure in children (parentally reported and verified with the children’s urine cotinine concentration) 6 months after the start of the intervention. The secondary outcome measures will also be analysed for differences between the intervention and control group. Dichotomous outcomes (stopping PS exposure [by elimination or reduction] in children [yes/no]) will be analysed with mixed logistic regression of PS exposure stopping status on treatment group, time and group by time interaction. The analysis will both evaluate the group difference at the primary time point, 6 months, and the group divergence over time. The analysis is an intention-to-treat analysis which includes all randomized families with at least one measurement. Continuous outcomes will be similarly analysed with mixed linear regression. Non-normality of such outcomes will be handled by data transformation, for example a logarithmic or square root transformation in case of severe positive skewness. Given randomized assignment, no confounding is expected except due to dropout, which is accommodated by using baseline variables as covariates and including all randomized children into the outcome analyses (intention-to-treat analysis) using the direct Likelihood approach of mixed regression, which requires no imputation of missing outcome values. The primary outcome eliminating or reducing PS exposure will be tested two-tailed with an alpha of 5%, but secondary outcomes with an alpha of 1% to prevent type I errors due to multiple testing.

### Ethics

The PREPASE study was reviewed and approved by the Medical Ethics Committee MUMC+, and is registered at the Dutch Trial Register (NTR2632). The study protocol is reviewed by the funding organization: the Dutch Lung Foundation (grant 3.4.08.047).

## Discussion

This manuscript describes the design of the PREPASE study, a RCT testing the effectiveness of an intervention program to prevent PS exposure in children with a high risk of asthma. The main strength of the PREPASE study is that it incorporates successful elements of previous interventions to prevent PS exposure in children into one program. If proven effective, the intervention can also be tested in other groups, such as children without high risk of asthma. We believe that the strongest component of our program is that parents receive individualized counselling in the comfort of their own homes. Furthermore, the MI approach offers parents the room to make their own decisions. Emphasis is put on empathy and positive reflections and reinforcements by the RA. However, there are some possible limitations of the study. Ideally, the best method to prevent all PS exposure in children is if parents quit active smoking. We can advise parents to quit smoking, but not force them. Parents are free to choose to smoke for example only in a room where the child does not enter or outside. The parents are motivated to make goals that they believe they can adhere to, not what they think we expect them to do.

Furthermore, the information in the invitation letter concerning the aim of our study could also induce stopping behaviour in the control group. But due to ethical reasons we are obliged to provide all the participants some information of the study. In addition, getting parents to participate in this study can be very challenging. Currently, there is much attention in the media about PS exposure and its negative health effects. Parents might not want to participate in the PREPASE study because of feelings of shame or embarrassment. Consequently, this can lead to the participation of only parents who are highly motivated to prevent PS exposure in their children. However, if this is the case, we do not expect this to limit the generalizability of the study. If the intervention is successful and implemented in for example general practices, the chances are high that mostly motivated parents would seek help for stopping PS exposure in their children. Also, the prevalence of PS exposure is highest in socially deprived families. Selecting and communicating with such families can also be challenging. To minimize drop-outs as much as possible, all parents receive a reminder letter one month before the measurement. Furthermore, all children receive a small gift after each measurement, and the families receive a financial incentive after completion of the study.

## Conclusion

Various programs have been tested and have shown some effect to decrease PS exposure in children. However, there is still room for improvement. As children with a high risk of asthma are at increased risk of developing respiratory complaints such as wheezing and asthma due to PS exposure, effective programs are needed to prevent PS exposure in this group of children. The PREPASE study aims at testing such an intervention program that will not only have important beneficial health effects for children with higher risk of asthma, but is also of great importance for our society.

## Competing interests

All authors state that they have nothing to declare.

## Authors’ contributions

SH, IM, CS, and ED developed the study. GvB, JM, FF, SKH advised on the design of the study. SH, IM, GvB and ED helped to draft the manuscript. JM, FF, SKH and CP were involved in revising the manuscript. All authors read and approved the final manuscript.

## Pre-publication history

The pre-publication history for this paper can be accessed here:

http://www.biomedcentral.com/1471-2458/13/177/prepub

## Supplementary Material

Additional file 1 Supplement questionnaires specifically developed for the PREPASE study. Click here for file
