# Supplementary material for: A motivational interviewing intervention to PREvent PAssive Smoke Exposure (PREPASE) in children with a high risk of asthma: design of a randomised controlled trial
Source: BMC Public Health. 2013 Feb 27;13:177. doi: 10.1186/1471-2458-13-177 (PMC3599824; doi:10.1186/1471-2458-13-177)
Supplement: Additional file 1 — Supplement questionnaires specifically developed for the PREPASE study. [file 1471-2458-13-177-S1.doc]

**Supplement questionnaires specifically developed for the PREPASE study**

Recruitment questionnaires:

1. Questionnaire A
2. Questionnaire B (non-response)
3. Electronic questionnaire for recruitment via schools

Questionnaires used in the randomized control trial:

1. Questionnaire used at baseline (t0) and 12 months (t12) after baseline measurement
2. Questionnaire used 3, 6, and 9 months (t3, t6, t9) after baseline measurement
3. Process evaluation questionnaire (only in the intervention group)

**QUESTIONNAIRE A**

**General questions**

1. Date of completion: _ _ - _ _ - 20_ _ (dd/mm/yyyy)

2. Child birth date: _ _ - _ _ - _ _ _ _ (dd/mm/yyyy)

3. What is the sex of your child?

| - Male | - Female |
| --- | --- |

4. What is the relationship of you and your partner to your child?

|  | **Yourself** | **Your partner** |
| --- | --- | --- |
| Biological mother |  |  |
| Biological father |  |  |
| Stepmother |  |  |
| Stepfather |  |  |
| Other, namely: | - …………………………. | - …………………………. |
| Not applicable |  | - *(I do not have a partner)* |

5. What are the birth dates of you and your partner?

Yourself Your partner

_ _ - _ _ - _ _ _ _ (dd/mm/yyyy) _ _ - _ _ - _ _ _ _ (dd/mm/yyyy)

- Not applicable *(I do not have a partner)*

6. a. Do you have more children?

| - Yes | - No 🡪 if ‘No’ go to question 7 |
| --- | --- |

*b. Record for every other child their birth date. Also specify if this child is the biological brother/sister of the child mentioned at question 2 and whether he/she lives at home. (*With biologically we mean from the same father and mother.)

| **Birth date *(dd/mm/yyyy)*** | **Relation brother/sister** | **Lives at home?** |
| --- | --- | --- |
| _ _ - _ _ - _ _ _ _ | - Biological - Half - Other | - Yes - No   Etc. |

1. *Which situation does currently apply for you and your partner? More answers are* possible.

|  | **Yourself** | **Your partner** |
| --- | --- | --- |
| Working outdoors, 20 hours/more per week |  |  |
| Working indoors, less than 20 hours per week |  |  |
| Looking for work |  |  |
| Incapacitated |  |  |
| Working indoors (household) |  |  |
| Studying |  |  |
| Other, namely: | - ………………… | - *…………………* |
| Not applicable |  | - *(I do not have a partner)* |

1. What is your highest level of education? And your partners’?

|  | **Yourself** | **Your partner** |
| --- | --- | --- |
| Primary school |  |  |
| Lower vocational education |  |  |
| General secondary education |  |  |
| Middle vocational education |  |  |
| Senior general secondary education, pre-university |  |  |
| Higher vocational education |  |  |
| University, academic |  |  |
| Other, namely: | - ………………… | - ………………… |
| Not applicable |  | - *(I do not have a partner)* |

1. In which country were you and your partner born?

|  | **Yourself** | **Your partner** |
| --- | --- | --- |
| Netherlands |  |  |
| Suriname |  |  |
| Turkey |  |  |
| Morocco |  |  |
| Netherlands Antilles |  |  |
| Other, namely: | - ………………… | - ………………… |
| Not applicable |  | - *(I do not have a partner)* |

**Questions 10 – 47 are about the health of your child**

*(Questions 10 - 16: Questionnaire on Eczema (Module 1.4 obtained from the International Study of Asthma and Allergies in Childhood (ISAAC) phase II )*

1. Has your child ever had an itchy rash which was coming and going for at least six months?

| - Yes | - No 🡪 if ‘No’ go to question 16 |
| --- | --- |

11. Has your child had this itchy rash at any time in the last 12 months?

| - Yes | - No 🡪 if ‘No’ go to question 16 |
| --- | --- |

12. Has this itchy rash at any time affected any of the following places: the folds of the elbows, behind the knees, in front of the ankles, under the buttocks, or around the neck, ears or eyes?

| - Yes | - No |
| --- | --- |

1. At what age did this itchy rash first occur?

| - - Under 2 years   - Age 2-4 years   - Age 5 or more |
| --- |

1. Has this rash cleared completely at any time during the last 12 months?

| - Yes | - No |
| --- | --- |

1. In the last 12 months, how often, on average, has your child been kept awake at night by this itchy rash?

| - Never - Less than one night per week - One or more nights per week |
| --- |

1. Has your child ever had eczema?

| - Yes | - No |
| --- | --- |

*(Questions 17, 19 - 23, 26: Questionnaire on Wheezing (Module 1.2 ISAAC phase II ))*

17. Has your child ever had wheezing or whistling in the chest at any time in the past?

| - Yes | - No 🡪 if ‘No’ go to question 23 |
| --- | --- |

*18. In which period of his/her life has your child had wheezing or whistling in the chest? More answers are* possible.

| - In the first year of life - In the second year of life - In the third year of life - In the fourth year of life - In the fifth year of life - Other, namely ……………. |
| --- |

19. In the last 12 months, has your child had wheezing or whistling in the chest?

| - Yes | - No 🡪 if ‘No’ go to question 23 |
| --- | --- |

20. How many attacks of wheezing has your child had in the last 12 months?

| - None - 1-3 - 4-12 - More than 12 |
| --- |

21. In the last 12 months, how often, on average, has you child’s sleep been disturbed due to wheezing?

| - Never - Less than one night per week - One or more nights per week |
| --- |

22. In the last 12 months, has wheezing ever been severe enough to limit your child’s speech to only one or two words at a time between breaths?

| - Yes | - No |
| --- | --- |

23. In the last 12 months, has your child’s chest sounded wheezy during or after exercise?

| - Yes | - No |
| --- | --- |

24. Has your child ever had shortness of breath?

| - Yes | - No 🡪 if ‘No’ go to question 26 |
| --- | --- |

25. Has your child ever had shortness of breath in the past 12 months?

| - Yes | - No |
| --- | --- |

26. Has your child ever had asthma (physician diagnosed)?

| - Yes | - No |
| --- | --- |

*(Question 27: Asthma management (Module 2.2 ISAAC phase II ))*

27. a. In the past 12 months, has your child used any medicines, pills, puffers, or other medication for wheezing or asthma?

| - Yes | - No 🡪 if ‘No’ go to question 28 |
| --- | --- |

b. Please name the medication(s)

| **Medicine** | **How often? (please circle one or both)** | |
| --- | --- | --- |
| ……………… | When wheezy | Regularly (every day for at least two months of the year) |
| ……………… | When wheezy | Regularly (every day for at least two months of the year) |

*(Question 28: Questionnaire on Wheezing (Module 1.2 ISAAC phase II ))*

28. In the last 12 months, has your child had a dry cough at night, apart from a cough associated with a cold or chest infection?

| - Yes | - No 🡪 if ‘No’ go to question 30 |
| --- | --- |

29. In the last 12 months, how often, on average, has you child’s sleep been disturbed due to coughing at night, apart from cough associated with a cold or chest infection?

| - Never - Less than one night per week - One or more nights per week |
| --- |

*(Questions 30-35: Questionnaire on rhinitis (Module 1.3 ISAAC phase II ))*

30. Has your child ever had a problem with sneezing or a runny or blocked nose, when he/she did not have a cold or the flu?

| - Yes | - No 🡪 if ‘No’ go to question 35 |
| --- | --- |

31. In the past 12 months, has your child had a problem with sneezing or a runny or blocked nose when he/she did not have a cold or the flu?

| - Yes | - No 🡪 if ‘No’ go to question 35 |
| --- | --- |

32. In the past 12 months, has this nose problem been accompanied by itchy-watery eyes?

| - Yes | - No |
| --- | --- |

33. In which of the past 12 months did this nose problem occur*? More answers are possible.*

| - January | - May | - September |
| --- | --- | --- |
| - February | - June | - October |
| - March | - July | - November |
| - April | - august | - December |

34. In the past 12 months, how much did this nose problem interfere with your child’s daily activities?

| - Not at all - A little - A moderate amount - A lot |
| --- |

35. Has your child ever had hay fever?

| - Yes | - No |
| --- | --- |

36. In the past 12 months, has your child been diagnosed by a physician with one or more of the following health problems? If yes, has your child received prescribed medications and was he/she admitted in the hospital because of the health problem?

Please give an answer for each of the following health problems:

|  | **Physician diagnosed?** | | **Prescribed medication?** | | **Admitted in the hospital?** | |
| --- | --- | --- | --- | --- | --- | --- |
| Hay fever | - Yes | - No | - Yes | - No | - Yes | - No |
| Flu or severe cold | - Yes | - No | - Yes | - No | - Yes | - No |
| Throat infection | - Yes | - No | - Yes | - No | - Yes | - No |
| Middle ear infection | - Yes | - No | - Yes | - No | - Yes | - No |
| Sinusitis | - Yes | - No | - Yes | - No | - Yes | - No |
| Bronchitis | - Yes | - No | - Yes | - No | - Yes | - No |
| Pneumonia | - Yes | - No | - Yes | - No | - Yes | - No |

37. Did your child ever had other respiratory tract infections apart from those mentioned in question 36?

| - Yes, namely ……………. - No |
| --- |

38. In the past 12 months, how often did your child had a respiratory tract infection (such as, flu, severe cold, throat infections, middle ear infection, sinusitis, bronchitis or pneumonia) whereby you had to consult a physician?

| - Never - 1-2 times - 3-5 times - 6 times or more |
| --- |

39. a. Does your child get vitamin supplements?

| - Yes | - No 🡪 if ‘No’ go to question 40 |
| --- | --- |

b. Which vitamin supplements does your child get? Is this daily, occasionally or never?

|  | **Daily** | **Occasionally** | **Never** |
| --- | --- | --- | --- |
| Vitamin A |  |  |  |
| Vitamin B |  |  |  |
| Vitamin C |  |  |  |
| Vitamin D |  |  |  |
| Vitamin E |  |  |  |
| Omega 3 |  |  |  |
| Other:………… |  |  |  |

40. a. At how many weeks pregnancy was your child born? _ _ weeks

b. What was the birth weight of your child? _ _ _ _ grams

41. a. Did your child suffered from any health problems while your were pregnant with or gave birth to him/her?

| - Yes | - No 🡪 if ‘No’ go to question 42 |
| --- | --- |

b. Which health problems did your child suffered while you were pregnant with or gave birth to him/her? *More answers are possible.*

| - Growth retardation during pregnancy - Infections - Lack of oxygen - Other: …………………………………… |
| --- |

42. a. Was your child ever breastfed?

| - Yes: - Less than 6 months, namely _ _ weeks - 6 -12 months - More than 12 months | - No 🡪 if ‘No’ go to question 43 |
| --- | --- |

b. For how long was your child breastfed without adding other foods or juices?

| - Less than 2 months - 2 - 4 months - 5 - 6 months - More than 6 months |
| --- |

43. Did you or your partner smoke during or after the pregnancy of your child?

|  | **Yourself** | **Your partner** |
| --- | --- | --- |
| **During the pregnancy** | - Yes | - Yes |
|  | - No | - No |
|  |  |  |
| **After the pregnancy** | - Yes | - Yes |
|  | - No | - No |

44. a. Did a physician ever diagnose an inherent abnormality of the hart and/or lungs of your child?

| - Yes | - No 🡪 if ‘No’ go to question 45a |
| --- | --- |

b. Which inherent abnormality of the hart and/or lungs of your child has been diagnosed? ………………………………

45. a. Has your child been diagnosed with a syndrome by a physician?

| - Yes | - No 🡪 if ‘No’ go to question 46 |
| --- | --- |

b. Which syndrome has been diagnosed in your child? ………………………………

46. a. During the ages of 0 through 4 years, did your child ever go to a form of child care facility?

| - Yes | - No 🡪 if ‘No’ go to question 47 |
| --- | --- |

b. Which type of child care facility did your child attend?

| - Grandparent(s) - Host parents - Day care, nursery - Other: …………………………………… |
| --- |

47. For each health complaint below, please specify if your child has a biological relative with one of these complaints. Also, indicate whether this has been diagnosed by a physician.

| **Asthma family member?** (more answers are possible) | **Diagnosed by a physician?** | |
| --- | --- | --- |
| - No family member - Father - Mother - Biological sibling(s) - Half sibling(s) - Other:……………….. | - Yes - Yes - Yes - Yes - Yes | - No - No - No - No - No |
| **Eczema family member?** (more answers are possible) | **Diagnosed by a physician?** | |
| - No family member - Father - Mother - Biological sibling(s) - Half sibling(s) - Other:……………….. | - Yes - Yes - Yes - Yes - Yes | - No - No - No - No - No |
| **Hay fever family member?** (more answers are possible) | **Diagnosed by a physician?** | |
| - No family member - Father - Mother - Biological sibling(s) - Half sibling(s) - Other:……………….. | - Yes - Yes - Yes - Yes - Yes | - No - No - No - No - No |

**The following questions refer to your smoking habits**

*(Questions 48-61: Measurement instrument for research on smoking and smoking cessation )*

1. Do you sometimes smoke?

| - Yes, daily 🡪 go to question 53 - Yes, sometimes 🡪 go to question 53 - No, I am taking a quit attempt now 🡪 go to question 49 - No, not at all, or I stopped smoking more than 6 months ago 🡪 go to question 66 |
| --- |

1. How long has it been that you stopped smoking?

| - Less than 1 week, namely _ days - Less than 1 month, namely _ weeks - More than 1 month, but less than 6 months, namely _ months |
| --- |

1. Have you smoked since you stopped smoking?

| - No, not one puff - Yes, 1-5 cigarettes - Yes, more than 5 cigarettes |
| --- |

1. Did you persevere not to smoke for 24 hours or more at this stop attempt?

| - Yes, _ _ times - No |
| --- |

1. Have you used any aid or methods to stop smoking since you quit smoking?

More answers are possible.

| - No, no aid used - No-smoking course or group therapy - Nicotine gum - Nicotine band-aid - Nicotine pastilles - Nicotine microtabs (tablet for under the tong) - Zyban (bupropion) - Speaking to GP about smoking cessation - Acupuncture - Laser therapy - Telephone helpline - Folder - Book - Other: …………………………………. |
| --- |

**Here are a few questions about your smoking habit. If you are presently smoking, the following questions are related to your current smoking habit. If you are currently attempting to quit smoking, the following questions are related to the period when you were smoking.**

1. Which and how many of the following tobacco products do you smoke on average per day?

More answers are possible.

| - _ _ cigarettes per day - _ _ roll-ups per day - _ _ cigars/cigarillo’s per day - _ _ pipe per day |
| --- |

1. Have you smoked one or more cigarettes (roll-ups, cigars, pipe) over the past 24 hours?

| - Yes | - No |
| --- | --- |

1. Have you smoked one or more cigarettes (roll-ups, cigars, pipe) over the past 7 days?

| - Yes | - No |
| --- | --- |

*(Question 56: the Transtheoretical Model of Change )*

1. Are you planning to stop smoking in the future?

| - Not applicable, I am attempting to quit now - Yes, within 1 month - Yes, within 6 months, not the coming month - Yes within 1 year, , but not the coming 6 months - Yes, within 5 years - Yes, but not within 5 years - No, not planning to quit |
| --- |

*(Question 57 -61: Fagerstöm Test for Nicotine Dependence )*

1. How soon after you wake up do you start smoking your first cigarette?

| - Within 5 minutes - 6-30 minutes - 31-60 minutes - After 60 minutes |
| --- |

1. Do you find it difficult to refrain from smoking in places where it is forbidden (e.g. cinema, school, hospital, public transportation)?

| - Yes | - No |
| --- | --- |

1. Which cigarette would you hate most to give up?

| - The first one in the morning - All others (doesn’t matter which one) |
| --- |

1. Do you smoke more frequently during the first hours after waking than during the rest of the day?

| - Yes | - No |
| --- | --- |

1. Do you smoke if you are so ill that you are in bed most of the day?

| - Yes | - No |
| --- | --- |

1. Do you smoke inside your house?

| - Yes 🡪 if “Yes” go to question 63a - No 🡪 if “No” go to question 64a |
| --- |

1. a. Where do you smoke inside your house?

*More answers are possible.*

| - Living room - Your own bedroom - Kids bedroom - Kitchen - Under the cooker hood - Hallway - Dining room - Attic - Restroom - Other rooms, namely: …………………………………. |
| --- |

b. Do you open a window or door when you smoke inside your house?

| - Yes | - No |
| --- | --- |

1. a. Where do you smoke outside your house?

*More answers are possible.*

| - Balcony - Yard - Car - Other, namely: …………………………………. |
| --- |

b. Do you open the window when smoking in the car?

| - Yes - No - Not applicable (because I don’t smoke in the car) |
| --- |

1. a. Do you smoke in the presence of your child?

| - Yes - No 🡪 if “No” go to question 66 |
| --- |

b. Where do you smoke in the presence of your child and does this happen always, often, occasionally, or never?

*Please give an answer for each situation.*

|  | **Always** | **Often** | **Occasionally** | **Never** |
| --- | --- | --- | --- | --- |
| Living room |  |  |  |  |
| Own bedroom |  |  |  |  |
| Kids bedroom |  |  |  |  |
| Kitchen |  |  |  |  |
| Cooker hood |  |  |  |  |
| Dining room |  |  |  |  |
| Hallway |  |  |  |  |
| Attic |  |  |  |  |
| Toilet/bathroom |  |  |  |  |
| Balcony |  |  |  |  |
| Yard |  |  |  |  |
| Car |  |  |  |  |
| Other: ……… |  |  |  |  |

c. Are you willing to take measures to prevent tobacco smoke exposure to your child?

| - Yes | - No 🡪 if ‘No’ go to question 66 |
| --- | --- |

d. Which difficulties do you expect when taking measures to prevent tobacco smoke exposure to your child? *More answers are possible.*

I expect…

| - no difficulties - to find it difficult for myself to not smoke inside the house - to find it difficult with the smoking of partner - to find it difficult with the smoking of families and/or visitors - to find it difficult with judgment or lack of understanding from family members and/or visitors - to find it difficult with other things, namely: ………………………………. |
| --- |

**The following questions are about the smoking habits of your partner. If you do not have a partner, please fill this in at question 66.**

**Partner: your current partner (not necessarily the biological father/mother of your child).**

1. Does your partner sometimes smoke?

| - Yes, daily 🡪 go to question 71 - Yes, sometimes 🡪 go to question 71 - No, he/she is taking a quit attempt now 🡪 go to question 67 - No, not at all, or he/she stopped smoking more than 6 months ago 🡪 go to question 84 - I do not have a partner 🡪 go to question 84 |
| --- |

*(Questions 67-83 are the same as questions 49-65, but referring to the smoking behavior of the partner.)*

**Additional general questions about smoking**

*84. In which areas of the house is smoking not allowed? More answers* are possible.

| - All - Living room - Bedroom partners - Bedroom child - Kitchen - Dinner table / room - Hallway - Attic - Toilet - Other, namely: …………………………………. |
| --- |

85. a. Apart from you or your partner, do other people smoke inside your house?

| - Yes | - No 🡪 if ‘No’ go to question 86 |
| --- | --- |

b. Apart from you or your partner, which other people smokes in your house? Can you give an indication on how frequent this occurs? *Please provide an answer for each category.*

|  | Daily | 4-6 times per wk | 1-3 times per wk | 1-2 times per wk | Occasionally (max. 3 times per month) | Never |
| --- | --- | --- | --- | --- | --- | --- |
| Grandparent(s) |  |  |  |  |  |  |
| Friends |  |  |  |  |  |  |
| Sitter |  |  |  |  |  |  |
| Family |  |  |  |  |  |  |
| Neighbor |  |  |  |  |  |  |
| Other child (sibling(s)) |  |  |  |  |  |  |
| Others:…….. |  |  |  |  |  |  |

86. On average, how many times is your child present in a room where others are smoking at that moment?

| - Never - Occasionally (max. 3 times per month) - 1-2 times per month - 1-3 times per week - 4-6 times per week - Every day |
| --- |

87. On average, how many times is your child present in a room where people have smoked, when your child was not present at that moment?

| - Never - Occasionally (max. 3 times per month) - 1-2 times per month - 1-3 times per week - 4-6 times per week - Every day |
| --- |

**“Since July 2008, smoking is prohibited in public areas in the Netherlands.”**

88. Do you think that this measure is a good idea?

| - Yes | - No |
| --- | --- |

89. How has this affected you? ……………………………………………………….

90. Are you aware that your surroundings are also smoking with you when you are smoking at the moment?

| - Yes | - No 🡪 END questionnaire |
| --- | --- |

*91. Where/how did you receive information about passive smoking? More answers are possible.*

| - Internet - STIVORO - General practitioner - School - Other, namely:……………………………………. |
| --- |

**END of the Questionnaire**

References:

1. **International Study of Asthma and Allergies in Childhood. Phase II Modules**.

2. Mudde A.N. WMC, Kremers S., Vries de H.: **Meetinstrumenten voor onderzoek naar roken en stoppen met roken. *(Measurement instruments for research on smoking and smoking cessation)***, Tweede druk *(second edition)* edn. Den Haag, Nederland *(The Hague, Netherlands)*: STIVORO voor een rookvrije toekomst *(STIVORO for a smokefree future)*; 2006.

3. Fava JL, Velicer WF, Prochaska JO: **Applying the transtheoretical model to a representative sample of smokers**. *Addictive behaviors* 1995, **20**(2):189-203.

4. Heatherton TF, Kozlowski LT, Frecker RC, Fagerstrom KO: **The Fagerstrom Test for Nicotine Dependence: a revision of the Fagerstrom Tolerance Questionnaire**. *British journal of addiction* 1991, **86**(9):1119-1127.

**Questionnaire B**

1. Date of completion: _ _ - _ _ - 20_ _ (dd/mm/yyyy)

2. Child birth date: _ _ - _ _ - _ _ _ _ (dd/mm/yyyy)

3. What is the relationship of you and your partner to your child?

|  | **Yourself** | **Your partner** |
| --- | --- | --- |
| Biological mother |  |  |
| Biological father |  |  |
| Stepmother |  |  |
| Stepfather |  |  |
| Other, namely: | - …………………………. | - …………………………. |
| Not applicable |  | - (I do not have a partner) |

1. Do you and/or your partner smoke and does this happens in the presence of your child?

|  | **Current smoking** | **Smoking in the presence of your child** |
| --- | --- | --- |
| You | - Yes - No | - Yes - No |
|  |  |  |
| Partner | - Yes - No | - Yes - No |

1. Are you planning to stop smoking in the future?

| - Not applicable, I am attempting to quit now - Yes, within 1 month - Yes, within 6 months, not the coming month - Yes within 1 year, , but not the coming 6 months - Yes, within 5 years - Yes, but not within 5 years - No, not planning to quit |
| --- |

6. Where do you and/or your partner smoke in the presence of your child and does this happen always, often, occasionally, or never?

Please give an answer for each situation.

|  | **Always** | **Often** | **Occasionally** | **Never** |
| --- | --- | --- | --- | --- |
| Living room |  |  |  |  |
| Own bedroom |  |  |  |  |
| Kids bedroom |  |  |  |  |
| Kitchen |  |  |  |  |
| Cooker hood |  |  |  |  |
| Dining room |  |  |  |  |
| Hallway |  |  |  |  |
| Attic |  |  |  |  |
| Toilet/bathroom |  |  |  |  |
| Balcony |  |  |  |  |
| Yard |  |  |  |  |
| Car |  |  |  |  |
| Other: ……… |  |  |  |  |

7. Has your child ever had asthma (physician diagnosed)?

| - Yes | - No |
| --- | --- |

8. Has a biological parent or sibling of your child been diagnosed with asthma?

| - Yes, namely ……………………………………… - No |
| --- |

9. In the past 12 months, how often did your child had a respiratory tract infection (such as, flu, severe cold, throat infections, middle ear infection, sinusitis, bronchitis or pneumonia) whereby you had to consult a physician?

| - Never - 1-2 times - 3-5 times - 6 times or more |
| --- |

10. In the last 12 months, has your child had wheezing or whistling in the chest?

| - Yes | - No |
| --- | --- |

11. How many attacks of wheezing has your child had in the last 12 months?

| - None - 1-3 - 4-12 - More than 12 |
| --- |

*12. What is your reason(s) for not participating in the study? More answers are possible.*

| - No interest - Lack of time - You are currently moving - Your child does not have respiratory complaints - Your child has a lot of respiratory complaints, and the burden is heavy - Smoking is not allowed in your house - Your child has been exposed to tobacco smoke for years, and has never experienced complaints because of the tobacco smoke exposure - You have no reason / you do not want to give a reason - Other, namely:………………………………………………….. |
| --- |

**Questionnaire for Schools**

**General questions**

*The following questions from questionnaire A are asked: questions 1-4, 6a, 7-9.*

*Additionally the following questions are asked:*

- What is the first name of your child? …………………………

What is the last name of your child? …………………………

- What is the name of the school of your child? …………………………

In which group is your child? .

**Questions about the child’s health**

*The following questions from questionnaire A are asked: questions 17-29, 36-38, 40-45, 47*

**Questions about parental smoking habits**

*The following questions from questionnaire A are asked: questions 48, 53, 62, 65a (for both parents).*

**Questionnaire t0 & t12**

*In addition to all the questions from questionnaire A, the following questions are included in the questionnaires for the measurement at baseline and 12 months of follow up:*

**Home situation**

1. How many people, adults and children, are currently living in the house where your child is living (including your own child)?

_ _amount of people

2. a. Do you think that your child lives in an area with air pollution? *(Factory fumes, heavy traffic, etc.)*

| - Yes | - No |
| --- | --- |

b. If yes, please indicate to what extent

| - Many - Poor - A little |
| --- |

3. What is used for heating in the house where your child lives? *More answers are possible.*

| - CH gas - Electric - Gas heater - Wood-burning stove - Other, namely …………………………………… |
| --- |

1. What system is used for cooking in the home where your child lives?

| - - Electric or convection   - Gas   - Other, namely …………………………………… |
| --- |

1. Is the house where your child lives upholstered?

| - Yes, namely with:   - Curtain   - Carpeting   - Fabric vanes   - Other, namely …………………………………. - No |
| --- |

1. Is there mold or wet spots in the house where your child lives?

| - Yes | - No |
| --- | --- |

1. Were there any pets in your house these past 12 months?

| - Yes | - No 🡪 if ‘No’ go parental smoking behavior |
| --- | --- |

1. Were the pets present throughout the whole period in your home?

| - Yes | - No, namely:_ _ months |
| --- | --- |

1. *Which pets did you have at home the past 12 months? More answers are possible.*

| - Cat, amount: _ _ - Dog, amount: _ _ - Birds, amount: _ _ - Rodents (rabbit, hamster, cavia), amount: _ _ - Other, namely:   1…………………………………., amount: _ _  2…………………………………., amount: _ _ |
| --- |

1. *a. Which pets are currently present in the house where your child lives? More answers are possible.*

| - Cat, amount: _ _ - Dog, amount: _ _ - Birds, amount: _ _ - Rodents (rabbit, hamster, cavia), amount: _ _ - Other, namely:   1…………………………………., amount: _ _  2…………………………………., amount: _ _ |
| --- |

b. Were these pets indoors?

| - Yes | - No |
| --- | --- |

**Parental smoking behavior**

*Question 65 from questionnaire A is adjusted to:*

a. Do you smoke in the presence of your child?

| - Yes - No 🡪 if “No” go to the ‘e’ part of this question |
| --- |

*b. Where do you smoke in the presence of your child and does this happen always, often, occasionally, or never?* Please give an answer for each situation.

|  | **Always** | **Often** | **Occasionally** | **Never** |
| --- | --- | --- | --- | --- |
| Living room |  |  |  |  |
| Own bedroom |  |  |  |  |
| Kids bedroom |  |  |  |  |
| Kitchen |  |  |  |  |
| Cooker hood |  |  |  |  |
| Dining room |  |  |  |  |
| Hallway |  |  |  |  |
| Attic |  |  |  |  |
| Toilet/bathroom |  |  |  |  |
| Balcony |  |  |  |  |
| Yard |  |  |  |  |
| Car |  |  |  |  |
| Other: ……… |  |  |  |  |

c. How many cigarettes/roll-ups/cigars/pipe do you smoke on average per day inside your house while your child is present at that moment?

| - _ _ cigarettes per day - _ _ roll-ups per day - _ _ cigars/cigarillo’s per day - _ _ pipe per day - Not applicable (I don’t smoke in the house while my child is present at that moment) |
| --- |

d. How many cigarettes/roll-ups/cigars/pipe do you smoke on average per day **outside** your house while your child is present at that moment?

| - _ _ cigarettes per day - _ _ roll-ups per day - _ _ cigars/cigarillo’s per day - _ _ pipe per day - Not applicable (I don’t smoke outdoors while my child is present at that moment) |
| --- |

e How many cigarettes/roll-ups/cigars/pipe do you smoke on average per day inside your house while your child is absent at that moment?

| - _ _ cigarettes per day - _ _ roll-ups per day - _ _ cigars/cigarillo’s per day - _ _ pipe per day - I don’t smoke in the house while my child is absent at that moment) |
| --- |

**The following questions are about how you feel about preventing tobacco smoke exposure in your child**

1. Please mark to what extend you agree or disagree with the following statements about your own thoughts about avoiding tobacco smoke exposure in your child?
2. Avoiding tobacco smoke exposure in my child is important
3. Avoiding tobacco smoke exposure in my child protects his/her health
4. Avoiding tobacco smoke exposure in my child useful
5. If there is no smoking in the house, my child will not be smoking later (better example)
6. Not smoking in the house is also important when my child is absent
7. Smoking with a window/door open or under a cooker hood does not protect my child against tobacco smoke exposure (passive smoking)
8. If I ensure that my child is not exposed to tobacco smoke, I am satisfied with myself
9. If there is no more smoking in the house, I will miss the fun
10. Preventing tobacco smoke exposure in my child is my responsibility
11. If I forbid smoking in my house, fewer visitors will come to visit
12. Other people will find it uncomfortable when I ask them not to smoke in the presence of my child
13. Children are in my opinion especially vulnerable to tobacco smoke
14. To be well informed about the effects of tobacco smoke exposure in my child is important to me
15. I worry about the effects of tobacco smoke exposure in child

***Answering categories: totally disagree, disagree, neutral, agree, totally agree, I do not know***

1. Please mark to what extent you agree or disagree with the following statements about your thoughts on your surroundings and prevention of cigarette smoke exposure in my child?
2. My partner thinks there should be no smoking in the house
3. My partner thinks that there should be no smoking in the presence of our child
4. Other people who are important to me (family, friends) think that there should be no smoking in the presence of my child
5. Our GP thinks that there should be no smoking in the house
6. Most of the people in my environment do not smoke in the house
7. Most of the people with children in my environment do not smoke in the house

***Answering categories: totally disagree, disagree, neutral, agree, totally agree, I do not know, not applicable (I do not have a partner)***

1. Please mark to what extent you agree or disagree with the following statements about your willingness to prevent tobacco smoke exposure in your child and your confidence that it will work to avoid tobacco smoke exposure in your child?
2. I intent to prevent tobacco smoke exposure in my child
3. I intent to take measures in my house to prevent tobacco smoke exposure in my child
4. I intent to stop smoking in the house
5. I intent to stop smoking in the car
6. I am confident that I am able to make my house smoke free
7. I am confident that I am able to make my care smoke free
8. I am confident that I can protect my child against tobacco smoke exposure

***Answering categories: totally disagree, disagree, neutral, agree, totally agree, don’t know, not applicable (I do not smoke or I do not smoke in the house/car)***

1. Please mark to what extent you find it difficult / easy to prevent tobacco smoke exposure in your child?
2. To stop smoking in the house I think is
3. To stop smoking in the car I think is
4. To ask other people not to smoke in the presence of my child I find
5. To ask smokers to smoke outside and not in my house I thinks is
6. To take measures to ensure that my child is not exposed to tobacco smoke is

***Answering categories: very difficult, difficult, neutral, easy, very easy, don’t know, not applicable (I do not smoke or I do not smoke in the house/car)***

*The questions about parental smoking behavior and beliefs towards stopping tobacco smoke exposure in children are asked to both parents (if applicable).*

**Questionnaire t3/t6/t9**

*The questionnaire is the same as the t0 questionnaire, but the ISAAC questionnaire is completely excluded and the questions concerning the home situation.*

**Process evaluation questionnaire (intervention group)**

For the evaluation of this study, we would like to ask you to give your opinion concerning a number of issues.

This section is about the monthly interviews and guidance from your coach.

1. How satisfied are you about …
   1. The guidance of the coach at home?
   2. The content of the conversations?

***Answer categories: not at all satisfied, not satisfied, neutral, satisfied, completely satisfied***

1. The following statements refer to **the conversations**. Please mark to what extent you agree or disagree with each of the following statements?
   1. The interview duration (40-60 minutes) was sufficient.
   2. The conversations with the coach motivated me (even more) to reduce smoking around my child.
   3. There was sufficient attention placed on the difficulties I encountered in the prevention of tobacco smoke exposure in my child.
   4. The coach paid enough attention to me during the home visits.
   5. I felt understood by the coach during the home visits.
   6. I was given enough opportunities to ask questions during the home visits.
   7. During the home visits I felt that things were discussed which were important to me.
   8. I had the feeling that the coach took me seriously during the home visits.
   9. Discussing the pros and cons about tobacco smoke exposure in my child has helped me with my decision.
   10. Because of the conversations I am now more motivated to reduce smoking in the presence of my child.
   11. Because of the conversations, I am consciously smoking less.
   12. Making a quit plan with a date to quit has given me more confidence that I can prevent smoking in the presence of my child.
   13. The number of conversations was just right.
   14. The period between two successive conversations was good.

***Answer categories: strongly disagree, disagree, neutral, agree, totally agree***

1. The following statements refer to the quit plan. Please mark to what extent you agree or disagree with each of the following statements?
   1. I have made a quit plan to reduce tobacco smoke exposure in my child.
   2. Making a quit plan was useful.
   3. Making a cessation- plan with quit date has given me more confidence that I can prevent smoking in the presence of my child.
   4. To stop, I made ​​use of the quit plan in the workbook.
   5. I have planned a quit date to start my reducing tobacco smoke exposure in my child.
   6. I made my own plan, without making use of the quit plan of the program.
   7. I was encouraged by the coach to make a quit plan.
   8. The coach always referred back to my quit plan, during our meetings.

***Answer categories: strongly disagree, disagree, neutral, agree, totally agree, not applicable (I did not make use of a quit-plan)***

1. The following statements concern the workbook. Please mark to what extent you agree or disagree with each of the following statements?
   1. The workbook was informative.
   2. The information in the workbook was clear.
   3. The workbook was useful.
   4. We have paid sufficient attention to the tasks and questions in the workbook.
   5. I also used the workbook during my own time.
   6. I did not use the workbook.
   7. The workbook was extensive.
   8. The workbook should have been more appealing.

***Answer categories: strongly disagree, disagree, neutral, agree, totally agree***

1. The coach discussed the results of the urinary cotinine concentrations of your child with you. Could you please describe in your own words how you have experienced this?

……………………………………………………………………………………………………………………………………………………………………………………………………………………………………

1. The following statements concern the discussion of the urinary cotinine concentrations of your child. Please mark to what extent you agree or disagree with each of the following statements?
   1. Discussing the results of the urinary cotinine concentrations of my child was confrontational.
   2. Discussing the urinary cotinine concentrations has convinced me that tobacco smoke exposure is harmful to my child.
   3. Discussing the urinary cotinine concentrations has motivated me to prevent tobacco smoke exposure in my child.
   4. Discussions on the urinary cotinine concentrations were clear.

***Answer categories: strongly disagree, disagree, neutral, agree, totally agree***

1. Did you miss or have comments about the program for reducing smoking in the presence of your child?

……………………………………………………………………………………………………………………………………………………………………………………………………………………………………

1. Has a doctor (general practitioner, paediatrician, clinic doctor, and paediatrician) previously discussed with you the health effects of tobacco smoke exposure in your child and measures to reduce tobacco smoke exposure in your child?

| ❑ yes, namely ❑ the GP  ❑ the paediatrician  ❑ the well-baby clinic doctor  ❑ the school doctor  🡪 go to 9 |
| --- |
| ❑ No 🡪 go to 10 |

1. Can you describe in your own words the difference between the approach of the doctor and this new program?

……………………………………………………………………………………………………………………………………………………………………………………………………………………………………

1. According to you, should this program be offered to all parents who smoke to reduce tobacco smoke exposure in their child(ren)?

| ❑ Yes |
| --- |
| ❑ No |

1. May we approach you in the future in the context of this research if we have any questions?

| ❑ Yes |
| --- |
| ❑ No |

Space for comments: ………………………………………………………………………………………………………………………………………………………………………………………………………………………………………………………………………………………………………………………………………………………………………

**You have finished filling out the evaluation questionnaire. Thank you for your participation in this study!**
